# Licofelone, a Dual COX/LOX Inhibitor, Ameliorates Paclitaxel-Induced Mechanical Allodynia in Rats in a Cannabinoid Receptor-Dependent Manner

**DOI:** 10.3390/biomedicines12071545

**Published:** 2024-07-11

**Authors:** Willias Masocha, Esraa Aly, Aisha Albaloushi, Altaf Al-Romaiyan

**Affiliations:** Department of Pharmacology and Therapeutics, College of Pharmacy, Kuwait University, Safat 13110, Kuwait; e.abbas@ku.edu.kw (E.A.); a.slbaloushi@ku.edu.kw (A.A.); altaf.alromaiyan2@ku.edu.kw (A.A.-R.)

**Keywords:** dual COX/LOX inhibitor, chemotherapy-induced neuropathic pain, CB1 receptor, CB2 receptor, allodynia, molecular docking, rat model, indomethacin, minocycline, combination therapy, 1-trans-delta-9-tetrahydrocannabinol

## Abstract

The use of paclitaxel as a chemotherapeutic drug is limited by the development of dose-dependent paclitaxel-induced neuropathic pain (PINP). Recently, we observed that the combination of indomethacin plus minocycline (IPM) attenuates PINP in a mouse model in a cannabinoid (CB) receptor-dependent manner. Indomethacin inhibits cyclooxygenase (COX) activity, and minocycline inhibits 5-lipoxygenase (5-LOX) activity. Male Sprague Dawley rats with paclitaxel-induced mechanical allodynia were treated with indomethacin, minocycline, IPM combination, licofelone (a dual COX/LOX inhibitor), or their vehicles. AM251, a CB1 receptor antagonist, and AM630, a CB2 receptor antagonist, were administered before the IPM combination or licofelone. Mechanical allodynia was measured using a dynamic plantar aesthesiometer. Molecular docking was performed using CB-Dock2. Licofelone and IPM combination had antiallodynic effects, which were significantly higher than either indomethacin or minocycline alone. AM251 and AM630 blocked the antiallodynic effects of IPM combination and licofelone. Molecular docking showed that licofelone binds to both CB1 and CB2 receptors with a high affinity similar to the phytocannabinoid 1-trans-delta-9-tetrahydrocannabinol and the synthetic cannabinoid WIN 55,212-2. Licofelone inhibits COX and LOX and/or directly interacts with CB receptors to produce antiallodynic effects in a rat model of PINP. The findings further suggest that licofelone could be a therapeutic agent for managing PINP.

## 1. Introduction

Peripheral neuropathy develops in about 70% of cancer patients undergoing chemotherapy with drugs such as paclitaxel, of which a significant number of these patients develop painful neuropathy known as chemotherapy-induced neuropathic pain (CINP) [1,2]. The CINP normally presents in a glove and stocking manner in some of the patients with symptoms such as increased sensation to pain (hyperalgesia), pain experienced from non-painful stimuli (allodynia), and spontaneous sensations such as burning, shooting, numbness, spasm, and pricking. Paclitaxel-induced neuropathic pain (PINP) can seriously affect the quality of life of the patient [3] and necessitate dose reduction, a switch to less efficacious agents, or even cessation of treatment in severe cases [4,5].

Unfortunately, there are currently no drugs to prevent the development of CINP in patients [6]. In terms of treatment, there is a dearth of effective drugs to manage CINP. The 2020 American Society of Clinical Oncology (ASCO) guidelines only recommend duloxetine (Strength of recommendation: moderate) as a treatment for CINP [7]. Thus, there is a need to search for effective drugs to manage CINP.

Our previous studies show that co-administration of indomethacin plus minocycline (IPM) produces antihyperalgesic and antiallodynic effects in mice models of CINP, whereas the individual drugs have minimal activity against these models of neuropathic pain [8,9]. Indomethacin produces analgesia and anti-inflammatory activities principally by inhibiting cyclooxygenase (COX) activity and, consequently, prostaglandin synthesis [10,11]. Minocycline has been reported to inhibit 5-lipoxygenase (5-LOX) activity [12]. Co-administration of COX and 5-LOX inhibitors was demonstrated to have potentiated antinociceptive activity in models of acute pain [13]. Thus, we hypothesized that dual inhibition of COX and 5-LOX produces antihyperalgesic and antiallodynic effects in an animal model of CINP.

There are several dual COX/LOX inhibitors, such as flavocoxid, licofelone, and tepoxalin, that have been researched for the management of pain [14,15]. Licofelone was chosen for this study because it has been reported to have neuroprotective effects (reviewed by [16]). Licofelone completed phase III clinical trials for the management of osteoarthritis (OA) with mixed results [17,18]. It inhibits both COX and LOX almost to the same level and has antiallodynic effects against incisional pain in rats and no toxicity was observed with doses up to 300 mg/kg po [17,19]. Licofelone has a better pharmacodynamic effect and is well tolerated than NSAIDs such as indomethacin [17]. In one study, licofelone was found to attenuate mechanical hypersensitivity during the chronic phase of spinal cord injury [20]. Thus, it is plausible that a dual COX/LOX inhibitor such as licofelone could alleviate symptoms of CINP.

In this study, we evaluated the antiallodynic effects of licofelone on a rat model of PINP and compared its effects to that of the IPM combination. Since we previously observed that the IPM combination produced effects in a cannabinoid (CB) receptor-dependent manner [8,9], the role of CB receptors on the activity of licofelone was investigated using in vivo and molecular docking studies.

## 2. Materials and Methods

### 2.1. Animals

The animals used in this study, male Sprague Dawley (SD) rats (8 to 12 weeks old; 200–300 g, *n* = 137), were supplied by the Animal Resources Center at the Health Sciences Center (HSC), Kuwait University, Kuwait. They were group-housed (*n* = 4–8 per cage) in plastic cages (Conventional EU Type 3H Cage with height 180 mm and floor area 800 cm^2^) with sawdust bedding and kept in temperature-controlled (24 ± 1 °C) rooms with food (standard laboratory chow) and water ad libitum. All experiments were performed during the same period of the day (0800 to 1600 h) to exclude diurnal variations in pharmacological effects. The animals were handled in compliance with Directive 2010/63/EU of the European Parliament and the Council on the protection of animals used for scientific purposes. All animal experiments were approved by the Ethical Committee for the Use of Laboratory Animals in Teaching and Research, HSC, Kuwait University. At the end of each experimental period, rats were euthanized by carbon dioxide administration. The number of experimental groups and number of animals per experimental group are detailed in Table 1 below, and the number of animals used in each specific treatment group is detailed in Supplementary Table S1 and the figure legends. The animals used were from different litters; however, each group treatment comparison with control was drawn with animals from the same litter.

### 2.2. Administration of Paclitaxel to Induce Mechanical Allodynia

Paclitaxel (obtained from both Tocris, Bristol, UK, and Thermo Scientific Chemicals J62734.03, Ward Hill, MA, USA) was dissolved in a solution made up of 50% Cremophor EL and 50% absolute ethanol to a concentration of 6 mg/mL, stored at −20 °C and used within 14 days. This stock solution was then diluted in normal saline (NaCl 0.9%) to a final concentration of 3.2 mg/mL just before administration. Paclitaxel (8 mg/kg body mass), or its vehicle, was administered to rats intraperitoneally (i.p.) in a volume of 2.5 mL/kg on two alternate days. We have previously observed mechanical allodynia in SD rats using this treatment regimen [21].

### 2.3. Drug Administration

Licofelone was purchased from Santa Cruz Biotechnology (Dallas, TX, USA), indomethacin and minocycline from Sigma-Aldrich (St Louis, MO, USA), and AM251 (CB1 receptor antagonist) and AM630 (CB2 receptor antagonist) from Tocris (Bristol, UK). Licofelone was suspended in 1.5% carboxymethylcellulose in normal saline (0.9% NaCl). Minocycline was dissolved in phosphate-buffered saline, whereas indomethacin, AM251, and AM630 were dissolved in normal saline containing 5% ethanol, 5% Cremophor, and 5% DMSO. Licofelone and its vehicle were freshly prepared and administered by oral gavage at a volume of 4 mL/kg body mass. The other drugs and their vehicles were freshly prepared and administered, i.p., to rats at a volume of 1 mL/kg body mass.

The dose of licofelone 50 mg/kg was chosen based on the dose used in previous research with rat models of neuropathic pain [20]; other doses higher and lower than the chosen dose were used for a dose-response curve. The doses of indomethacin 10 mg/kg, minocycline 50 mg/kg, AM251 3 mg/kg, and AM630 3 mg/kg were chosen based on our previous research with mice [9,22] and other studies on rats [23,24,25,26]. The dose of minocycline 50 mg/kg was also similar to previous research with rat models of neuropathic pain [27,28].

For acute treatment of rats with established paclitaxel-induced allodynia, licofelone, indomethacin, minocycline, and the IPM combination were administered on day 7 or 9 after the first dose of paclitaxel. The CB receptor antagonists (AM251 and AM630) were administered 15 min before licofelone or the IPM combination. The drugs were tested on day 7 or 9 after paclitaxel treatment, time points when the rats had reached the nadir of the paclitaxel-induced reduction in paw withdrawal threshold to mechanical stimuli [21].

### 2.4. Assessment of Mechanical Allodynia

Mechanical allodynia was measured using the dynamic plantar aesthesiometer (Ugo Basile, Gemonio, Italy), as previously described [21,29]. Briefly, rats were left to habituate for at least 30 min inside plastic enclosures on top of a perforated platform before measuring the withdrawal threshold (grams) to an automated filament that exerted a linearly increasing force (2.5 g/s with a cut-off time of 20 s) on the hind paw.

### 2.5. Molecular Docking

The 3D structures of licofelone (PubChem CID 133021), WIN 55,212-2 (PubChem CID 5311501) and 1-trans-delta-9-tetrahydrocannabinol (THC, PubChem CID 16078) were downloaded from PubChem database in .sdf format (https://pubchem.ncbi.nlm.nih.gov/, last accessed on 24 June 2024). The 3D protein structure of the CB1 receptor (PDB id: 5XRA) and CB2 receptor (PDB id: 6KPC), both in the active conformation, were obtained from Protein Data Bank (PDB, https://www.rcsb.org/, last accessed on 11 June 2024), in .pdb format.

Molecular docking was performed using the publicly available online docking server CB-Dock2 from the Yang Cao lab (https://cadd.labshare.cn/cb-dock2/index.php, last accessed on 24 June 2024) using the structure-based blind docking option, as described previously [30].

### 2.6. Statistical Analyses

The Shapiro–Wilk test normality test was used to test the data for normality. Statistical analyses were performed using Student’s *t*-test, one-way analysis of variance (ANOVA) followed by Dunnett’s multiple comparison posttest, or two-way repeated measures ANOVA followed by Šídák’s or Tukey’s multiple comparisons tests using GraphPad Prism software (version 10.2). The differences were considered significant at *p* < 0.05. The results in the text and figures are expressed as the means ± S.E.M.

## 3. Results

### 3.1. Licofelone and IPM Alleviate Paclitaxel-Induced Mechanical Allodynia in a CB Receptor-Dependent Manner

**Figure 1:**
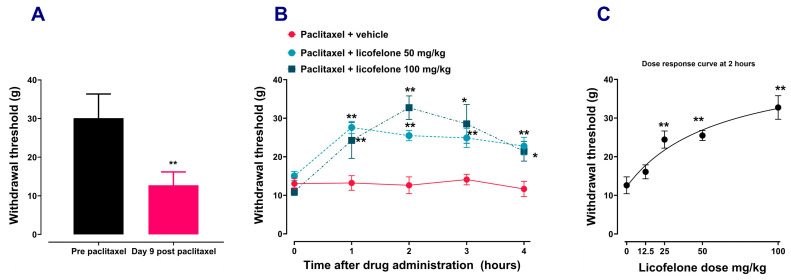
Licofelone alleviates established paclitaxel-induced mechanical allodynia. (**A**) Paw withdrawal thresholds at pre-paclitaxel administration (baseline) and on day 9 post-first administration of paclitaxel. Each bar represents mean ± S.E.M, *n* = 32, ** *p* < 0.01 compared to baseline (Paired Student’s *t*-test) (**B**) Time-response curve of withdrawal threshold at day 9 post-first administration of paclitaxel, before and after acute treatment with different doses of licofelone. Each point represents mean ± S.E.M, *n* = 5–8, * *p* < 0.05, ** *p* < 0.01 compared to the vehicle-treated group at the same time point after treatment (Two-way ANOVA followed by Šídák’s multiple comparisons test). (**C**) Dose-response curve of withdrawal threshold at day 9 post-first administration of paclitaxel, at 2 h after acute treatment with different doses of licofelone. Each point represents mean ± S.E.M, *n* = 5–8, ** *p* < 0.01 compared to the vehicle-treated group at the same time point after treatment (One-way ANOVA followed by Dunnett’s multiple comparison posttest).

Paclitaxel administration induced mechanical allodynia, similar to our previous results [21,29]. Rats with paclitaxel-induced allodynia (Figure 1A), treated with different doses of licofelone had time-dependent (Figure 1B) and dose-dependent (Figure 1C) increase in withdrawal threshold compared to those treated with vehicle (*p* < 0.01). Two-way repeated ANOVA showed there was a significant effect of treatment with licofelone 50 mg/kg [F (4, 44) = 5.707, *p* = 0.0009] and licofelone 100 mg/kg [F (4, 44) = 8.550, *p* < 0.0001] with time on the withdrawal thresholds compared to treatment with vehicle. One-way ANOVA showed that there was a dose-dependent increase in the withdrawal threshold after treatment with different doses of licofelone (12.5–100 mg/kg) compared to vehicle treatment (F (4, 27) = 13.3, *p* < 0.0001).

**Figure 2:**
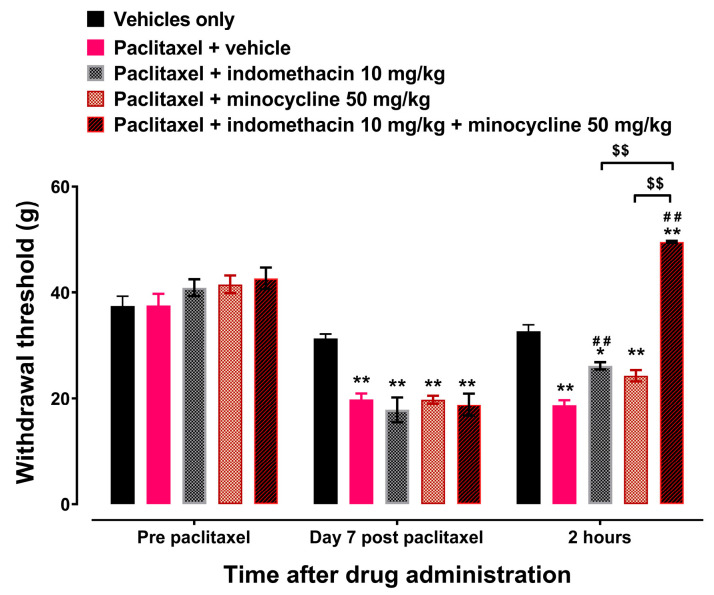
Indomethacin plus minocycline (IPM) combination alleviates established paclitaxel-induced mechanical allodynia. Withdrawal thresholds at pre-paclitaxel administration, on day 7 post-first administration of paclitaxel, and 2 h after acute treatment with indomethacin, minocycline, and IPM combination. Each point represents mean ± S.E.M, *n* = 8, * *p* < 0.05, ** *p* < 0.01 compared to the vehicle-treated group at the same time point after treatment, ## *p* < 0.01 compared to paclitaxel + vehicle at the same time point after treatment, $$ *p* < 0.01 between rats treated with indomethacin or minocycline alone and those treated IPM combination (Two-way ANOVA followed by Tukey’s multiple comparisons test).

Treatment with minocycline 50 mg/kg had no effect on the paclitaxel-induced decrease in withdrawal thresholds to mechanical stimuli at 2 h after treatment on day 7 after the first dose of paclitaxel compared to treatment with vehicle (Figure 2, *p* > 0.05). However, treatment with indomethacin 10 mg/kg or the IPM 60 mg/kg (indomethacin 10 mg/kg plus minocycline 50 mg/kg) combination significantly increased the withdrawal threshold at 2 h after treatment on day 7 after the first dose of paclitaxel compared to treatment with vehicle (Figure 2, *p* < 0.05). The IPM combination increased the withdrawal threshold significantly more than either indomethacin or minocycline alone (*p* < 0.01).

**Figure 3:**
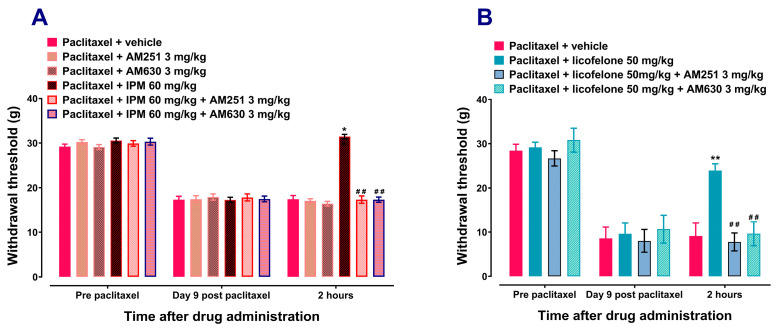
CB receptor antagonists prevent the antiallodynic effects of indomethacin plus minocycline (IPM) combination and licofelone. Withdrawal thresholds at pre-paclitaxel administration (baseline), on day 9 post-first administration of paclitaxel, and 2 h after acute treatment with the drugs. Effects of AM251, a CB1 receptor antagonist, and AM630, a CB2 receptor antagonist, on the antiallodynic effects of (**A**) IPM combination, *n* = 7–8, and (**B**) licofelone, *n* = 5, in rats with paclitaxel-induced mechanical allodynia at 2 h after administration. * *p* < 0.05, ** *p* < 0.01 compared to drug vehicle and ## *p* < 0.01 compared to IPM combination or licofelone at the same time point after treatment. (Two-way ANOVA followed by Šídák’s multiple comparisons test).

The CB1 receptor antagonist AM251 (3 mg/kg) or the CB2 antagonist AM630 (3 mg/kg) alone had no significant effect on rats with paclitaxel-induced mechanical allodynia. Treatment with the CB1 receptor antagonist AM251 (3 mg/kg) or the CB2 antagonist AM630 (3 mg/kg) alone had similar effects to vehicle treatment on the withdrawal thresholds to mechanical stimuli at 2 h after treatment (*p* > 0.05; Figure 3A). Both AM251 and AM630 significantly prevented the antiallodynic effects of IPM (Figure 3A) and licofelone (Figure 3B). AM251 and AM630 reduced the withdrawal threshold of IPM-treated rats, 31.443 ± 0.482 g for IPM 60 mg/kg alone compared to 17.313 ± 0.8178 g for IPM + AM251 (*p* < 0.01; Figure 3A) and 17.288 ± 0.580 g for IPM + AM630 (*p* < 0.01; Figure 3A), respectively. Similarly, AM251 and AM630 reduced the withdrawal threshold of licofelone-treated rats, 23.910 ± 1.510 g for licofelone 50 mg/kg alone compared to 7.780 ± 2.015 g for licofelone + AM251 (*p* < 0.01; Figure 3B) and 9.622 ± 2.690 g for licofelone + AM630 (*p* < 0.01; Figure 3B), respectively.

### 3.2. Licofelone, THC, and WIN 55,212-2 Interact with Similar CB1 and CB2 Receptor Cavities with Comparable Affinities

**Figure 4:**
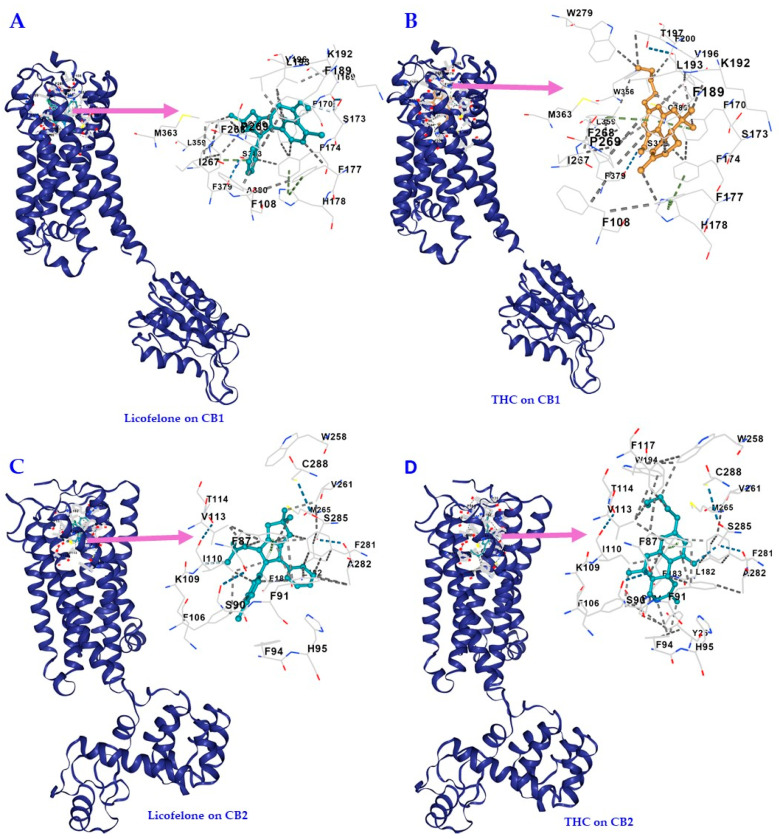
Licofelone and 1-trans-delta-9-tetrahydrocannabinol (THC) interact with similar CB1 and CB2 receptors’ cavities with comparable affinities. The best potential cavity regions on CB1 and CB2 receptors were detected by a structure-based approach. The interactive visualization for the 3D structure of (**A**) CB1 with licofelone bound to the cavity, (**B**) CB1 with THC bound to the cavity, (**C**) CB2 with licofelone bound to the cavity, and (**D**) CB2 with THC bound to the cavity.

The results of the ligand binding sites and poses of the molecular docking for licofelone on CB1 and CB2 receptors conducted on the CB-Dock2 server are shown in Table 2 and Table 3, respectively, as well as in Figure 4. The results of the molecular docking for THC on CB1 and CB2 receptors are shown in Table 4 and Table 5, respectively, as well as Figure 4. Table 2, Table 3, Table 4 and Table 5 show the results of the top five candidate cavities in terms of binding energy for the ligands and receptors, sorted by cavity volume. Table 6 shows the comparison of the best CB-Dock2 molecular docking Vina scores (kcal/mol) of licofelone and THC, as well as WIN 55,212-2, on CB1 and CB2 receptors.

The best binding energy for licofelone on CB1 was found to be −8.1 kcal/mol, which was less than that of THC and WIN 55,212-2 on the CB1 receptor (−11.2 kcal/mol and −10.6 kcal/mol, respectively), (Table 2, Table 4 and Table 6), suggesting high affinity and stable binding. The CB1 receptors’ cavity with the best binding energy for both licofelone, THC, and WIN 55,212-2 was the same (cavity volume [Å3] 3456, and center [x, y, z] −39, −168, 310). It has almost the same docking size ([x, y, z] 29, 21, 21 vs. 29, 22, 22 vs. 29, 23, 23) and shares almost all the amino acid contact residues (highlighted in red) the ligands bound to (Table 2 and Table 4, and Supplementary Table S2). That is:
Licofelone to CB1 C1 Chain A:PHE108 ILE169 PHE170 SER173 PHE174 ASP176 PHE177 HIS178 ASP184 PHE189 LYS192 LEU193 GLY194 VAL196 THR197 ALA198 PHE200 THR201 ILE267 PHE268 PRO269 TYR275 LEU276 TRP279 TRP356 LEU359 MET363 PHE379 ALA380 SER383 MET384 LEU385 CYS386 LEU387THC to CB1 C1 Chain A:PHE108 ILE169 PHE170 SER173 PHE174 ASP176 PHE177 HIS178 ARG182 ASP184 PHE189 LYS192 LEU193 VAL196 THR197 PHE200 ILE267 PHE268 PRO269 TRP279 TRP356 LEU359 MET363 PHE379 SER383 CYS386WIN 55,212-2 to CB1 C1 Chain A:Chain A: PHE108 VAL110 PHE170 SER173 PHE174 PHE177 HIS178 PHE189 LYS192 LEU193 VAL196 THR197 PHE200 ILE267 PHE268 PRO269 ILE271 TYR275 LEU276 TRP279 TRP356 LEU359 MET363 LYS376 PHE379 ALA380 SER383 CYS386

The best binding energy for licofelone on CB2 was found to be −9.4 kcal/mol., which was much better than CB1 (−8.1 kcal/mol) but less than that of THC and WIN 55,212-2 on the CB2 receptor (−11.2 kcal/mol and −13.5 kcal/mol, respectively), (Table 3, Table 5 and Table 6), all suggesting high affinity and stable binding. The CB2 receptors’ cavity with the best binding energy for both licofelone, THC, and WIN 55,212-2 was the same (cavity volume [Å3] 910 and center [x, y, z] 9, 2, −47), with almost the same docking size ([x, y, z] 21, 21, 21 vs. 22, 22, 22 vs. 23, 23, 23) and sharing almost all the amino acid contact residues (highlighted in red) the ligands bound to (Table 3 and Table 5, and Supplementary Table S3). That is:
Licofelone to CB2 C2 Chain A:TYR25 VAL86 PHE87 SER90 PHE91 PHE94 HIS95 PHE106 LYS109 ILE110 VAL113 THR114 PHE117 LEU182 PHE183 PRO184 TRP258 VAL261 MET265 LYS278 PHE281 ALA282 SER285 CYS288THC to CB2 C2 Chain A:TYR25 PHE87 SER90 PHE91 PHE94 HIS95 PHE106 LYS109 ILE110 GLY111 VAL113 THR114 PHE117 LEU182 PHE183 PRO184 LEU191 TRP194 TRP258 VAL261 MET265 PHE281 ALA282 SER285 CYS288WIN 55,212-2 to CB2 C2 Chain A:Chain A: TYR25 MET26 PHE87 SER90 PHE91 PHE94 HIS95 PHE106 LYS109 ILE110 GLY111 VAL113 THR114 PHE117 GLU181 LEU182 PHE183 PRO184 ILE186 TYR190 LEU191 TRP194 TRP258 VAL261 MET265 LYS278 PHE281 ALA282 SER285 CYS288

## 4. Discussion

The results of this study show that licofelone, a dual COX/LOX inhibitor, attenuates paclitaxel-induced mechanical allodynia in rats in a CB receptor-dependent manner, similar to the IPM combination. The effect of IPM in rats is similar to what we previously described in mice [8,9]. Treatment of rats with paclitaxel-induced mechanical allodynia with licofelone alleviated the allodynia in a time- and dose-dependent manner. Treatment with the IPM combination also alleviated the paclitaxel-induced mechanical allodynia, and the effect of IPM was higher than that of indomethacin or minocycline alone. Both the CB1 and CB2 receptor antagonists, AM251 and AM630, blocked the antiallodynic effects of IPM and licofelone. Molecular docking studies revealed that licofelone also interacts with CB1 and CB2 receptors on the same candidate cavities as the phytocannabinoid THC and the synthetic cannabinoid WIN 55,212-2, with similar conformational affinities.

In previous studies, we observed that the IPM combination had antinociceptive, antihyperalgesic, and antiallodynic effects in mice models of inflammatory and neuropathic pain, especially models of PINP [8,9,31]. The IPM combination had enhanced effects better than the individual drugs, indomethacin and minocycline alone, suggesting a synergistic effect [8]. In the current study, the IPM combination alleviated paclitaxel-induced mechanical allodynia in rats, similar to what we described previously [8]. This suggests a synergistic effect of indomethacin and minocycline when combined, taking into consideration the combination subthreshold, highest single agent, response additivity, and Bliss independence approaches [32]. Rats with paclitaxel-induced mechanical allodynia increased the withdrawal threshold when treated with indomethacin alone by about 60% and by about 20% when treated with minocycline alone, whereas treatment with IPM combination increased the withdrawal threshold by approximately 180%. Since indomethacin inhibits COX, which is mainly responsible for its antinociceptive activity, and minocycline inhibits the activity of LOX, which could partly be responsible for its limited activity, dual inhibition of COX/LOX could produce enhanced antiallodynic effects in models of CINP. Licofelone, a dual COX/LOX inhibitor [33], was evaluated and had antiallodynic effects in rats with paclitaxel-induced mechanical allodynia and increased the withdrawal threshold by 150%.

**Figure 5:**
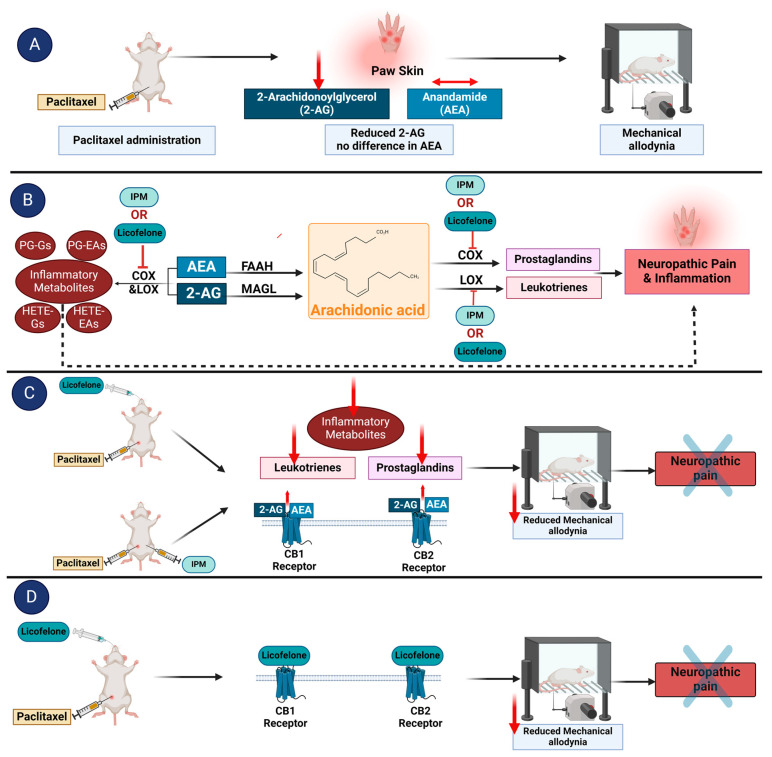
A scheme illustrating how licofelone and indomethacin plus minocycline (IPM) combination might produce antiallodynic effects in a CB receptor-dependent manner in a rodent model of paclitaxel-induced mechanical allodynia. (**A**) Treatment of rodents with paclitaxel reduces the endocannabinoid 2-AG without affecting AEA. The deficiency in 2-AG contributes to the development of mechanical allodynia [58]. (**B**) IPM and licofelone inhibit the activity of COX and LOX enzymes, reduce the conversion of 2-AG and AEA to inflammatory metabolites, and block the conversion of arachidonic acid to prostaglandins and leukotrienes. The inflammatory metabolites generated from endocannabinoids, prostaglandins, and leukotrienes could contribute to neuropathic pain symptoms and inflammation. (**C**) Treatment of rodents with paclitaxel-induced allodynia with either IPM or licofelone might result in increased levels of the endocannabinoids 2-AG and AEA, which produce antiallodynic activity via interactions with CB1 and CB2 receptors. The reduced inflammatory metabolites, prostaglandins, and leukotrienes after treatment with IPM or licofelone possibly contribute to their antiallodynic effects. (**D**) Molecular docking studies suggest that licofelone interacts directly with both CB1 and CB2 receptors to produce antiallodynic effects. Figure created by the authors using https://BioRender.com.

The dual COX/LOX inhibition could alleviate allodynia by reducing the levels of pro-inflammatory molecules (see Figure 5) or by increasing the levels of the endocannabinoids 2-arachidonoylglycerol (2-AG) and N-arachidonoylethanolamine (AEA, anandamide) since both COX and LOX are involved in the metabolism of endocannabinoids to produce inflammatory mediators [34,35,36,37]. If the antiallodynic effects of IPM combination and licofelone are due to an increase in the endocannabinoid levels, their antiallodynic effects would be blocked by CB receptors’ antagonists since the endocannabinoids produce their effects through interaction with CB receptors [38,39,40,41,42,43,44]. The antiallodynic effects of the IPM combination in rats were blocked by both CB1 and CB2 antagonists, similar to what we observed in mice with paclitaxel- and ddC-induced thermal hyperalgesia and mechanical allodynia [8,9]. Similar to IPM, the antiallodynic effects of licofelone were blocked by both CB1 and CB2 antagonists. Further biochemical studies are needed to ascertain whether licofelone or the IPM combination affects the levels of the endocannabinoids in the tissues.

Since the antiallodynic effects of licofelone were blocked by CB receptor antagonists, there is also a probability that licofelone binds to and has a direct effect on CB receptors (Figure 5). Molecular docking studies using the CB-Dock2 server revealed that licofelone binds to cavities in the CB1 and CB2 receptors with high affinity (−8.1 kcal/mol and −9.4 kcal/mol, respectively), suggesting a higher affinity for CB2 receptors compared to CB1 receptors. More interestingly, licofelone interacted with the same cavities as the phytocannabinoid THC and the synthetic cannabinoid WIN 55,212-2 with comparable conformational affinity (−11.2 kcal/mol and −10.6 kcal/mol for CB1 and −11.5 kcal/mol and −13.5 kcal/mol for CB2, respectively) and interacted with similar amino acid residues. The interaction of licofelone, THC, and WIN 55,212-2 with the following amino acid residues at the binding site of CB1 receptor (5XRA), PHE170, PHE174, PHE177, LEU193, THR197, TRP279, PHE379, and SER383 found in our study was also described for THC in other previous studies [45,46]. In addition, the interaction of licofelone, THC, and WIN 55,212-2 with the following amino acids at the binding site of CB2 receptor (6KPC), TYR25, HIS95, and LEU182, found in our study, has also been described for other CB2 agonists, such as AM-12033 in other previous studies [47]. According to El-Atawneh and Goldblum, CB2 receptor ligands are considered successfully docked if they interact with CB2 receptor (6KPC) through “hydrogen bonding with LEU182 and SER285, and Van der Waals (VDW) interactions with the following: TYR25, PHE87, PHE91, PHE94, ILE110, PHE183, TYR190, LEU191, TRP194, LEU262, MET265, PHE281” [48]. Interestingly, licofelone, THC, and WIN 55,212-2 had the described interactions with those amino acids when docked to 6KPC except for only four amino acid residues TYR190, LEU191, TRP194, and LEU262 for licofelone, two amino acid residues TYR190, and LEU262 for THC, and one amino acid residue LEU262 for WIN 55,212-2 and thus licofelone, THC and WIN 55,212-2 can be considered successfully docked to the CB2 receptor. The binding to similar residues by licofelone, THC, and WIN 55,212-2 in our studies and previous studies using different molecular docking software or servers confirm that the molecules highly likely bind to these cavities. However, the findings of the molecular docking need to be verified by in vitro or ex vivo ligand binding assays [49,50,51].

CB1 and CB2 receptors are G protein-coupled receptors (GPCR), which, through interaction with Gi/o proteins, inhibit calcium channels, activate potassium channels, inhibit adenylate cyclase activity, and activate mitogen-activated protein kinases [42,52,53]. Activation of CB receptors either by endocannabinoids or exogenous cannabinoids has been shown to produce antinociceptive and antiallodynic effects [9,52,54,55,56,57,58]. Activation of CB1 receptors in the brain, spinal cord, peripheral sensory neurons, or immune cells produces analgesic effects [55]. CB1 receptors reduce neuronal excitability and act on presynaptic neurons to inhibit the release of neurotransmitters and thus have a modulatory effect and reduce the activity of afferent input of peripheral nociceptors [55]. In the CNS, activation of CB1 receptors modulates the descending pain pathway and the affective/emotional aspects of pain to produce analgesia [55]. CB2 receptor activation reduces inflammation and pain without psychotropic effects, most likely through effects on immune cells, microglia, and astrocytes, altering cytokine expression and secretion [49]. Licofelone, through direct or indirect activation of CB1 and CB2 receptors, could alleviate pain, including neuropathic pain, as demonstrated by its ability to alleviate paclitaxel-induced mechanical allodynia.

Figure 5 summarizes how IPM combination and licofelone could produce their antiallodynic effects in rats with paclitaxel-induced allodynia.

## 5. Limitations

One of the major limitations of this study was that the model of paclitaxel-induced allodynia we established [21,29] and used in this study had only male rats instead of both sexes or only female rats as recommended by the Sex, Gender, and Pain Special Interest Group of the International Association for the Study of [59]. In the future, we will characterize paclitaxel-induced mechanical allodynia in female rats and utilize them to evaluate the effects of analgesic drugs. However, other studies using Sprague Dawley rats did not find significant sex differences in terms of paclitaxel-induced mechanical hypersensitivity (allodynia or hyperalgesia) [60,61,62] and response to analgesic drugs such as ketamine and morphine [61]. Another limitation is that we used only one dose of IPM combination derived from our previous studies with mice [8,9]. Although mice have a generally faster metabolic rate than rats, the drugs used for analgesia in these animals have overlapping dose ranges, although some have a lower dose mg/kg for rats compared to mice [63,64,65]. The IPM combination alleviated paclitaxel-induced mechanical allodynia in rats similar to mice. However, the effects of lower doses of IPM need to be studied in rats.

## 6. Conclusions

The findings of this study show that the IPM combination and licofelone, a dual COX/LOX inhibitor, alleviated paclitaxel-induced allodynia in a CB1 and CB2 receptor-dependent manner. The molecular docking studies suggest that licofelone has a high affinity for CB1 and CB2 receptors, similar to the phytocannabinoid THC and the synthetic cannabinoid WIN 55,212-2, and thus could produce antiallodynic effects through direct interaction with the CB receptors. However, the possibility that licofelone can produce antiallodynic effects by inhibiting COX and LOX, increasing endocannabinoids, and reducing pronociceptive and inflammatory metabolites is also there and warrants further research.

## Figures and Tables

**Table 1:** Experimental groups and the number of animals per experimental group.

Group	Nature of Experiment	Number of Animals per Group
Group 1	Effects of licofelone on paclitaxel-induced mechanical allodynia.	32
Group 2	Effects of indomethacin plus minocycline combination (IPM) on paclitaxel-induced mechanical allodynia.	40
Group 3	Effects of cannabinoid receptor antagonists on indomethacin plus minocycline combination (IPM)’s antiallodynic activities	45
Group 4	Effects of cannabinoid receptor antagonists on licofelone’s antiallodynic activities	20

**Table 2:** Molecular docking results of licofelone on CB1 receptor obtained from the CB-Dock2 server.

CurPocket ID	Vina Score (kcal/mol)	Cavity Volume (Å3)	Center (x, y, z)	Docking Size (x, y, z)	Contact Residues
C1	−8.1	3456	−39, −168, 310	29, 21, 21	Chain A: PHE108 ILE169 PHE170 SER173 PHE174 ASP176 PHE177 HIS178 ASP184 PHE189 LYS192 LEU193 GLY194 VAL196 THR197 ALA198 PHE200 THR201 ILE267 PHE268 PRO269 TYR275 LEU276 TRP279 TRP356 LEU359 MET363 PHE379 ALA380 SER383 MET384 LEU385 CYS386 LEU387
C2	−8.1	889	−41, −111, 245	21, 21, 21	Chain A: SER1010 THR1011 THR1012 ASN1014 THR1015 SER1058 THR1059 TRP1060 GLY1061 ASP1062 CYS1093 GLY1094 ASP1095 TRP1098 TYR1100 GLY1128 ASP1129
C3	−7.3	881	−46, −138, 299	31, 21, 21	Chain A: ARG150 PRO151 SER152 TYR153 HIS154 PHE155 ILE156 LEU209 ALA210 ALA211 ASP213 ARG214 TYR224 VAL228 THR229 ARG230 ALA233 PHE237 TYR294 THR344 ILE396 TYR397 ARG400 SER401 LYS402 ASP403
C4	−6.3	395	−41, −135, 285	21, 21, 21	Chain A: ARG214 SER217 ILE218 HIS219 PRO221 MET295 ILE297 LEU298 TRP299 LYS300 ALA301 HIS302 HIS304 ALA305 MET337 ASP338 LEU341 ALA342 LYS343 VAL346
C5	−5.5	236	−42, −132, 273	21, 21, 21	Chain A: TRP299 LYS300 HIS302 SER303 HIS304 ALA305 VAL306 ALA1002 LYS1003 ALA1029 GLY1030 TYR1031 GLU1032 ASP1034 GLU1048 GLY1049 PHE1050 ASP1051 LEU1052 ARG1086 LYS1087 ILE1148 MET337

**Table 3:** Molecular docking results of licofelone on CB2 receptor obtained from the CB-Dock2 server.

CurPocket ID	Vina Score (kcal/mol)	Cavity Volume (Å3)	Center (x, y, z)	Docking Size (x, y, z)	Contact Residues
C1	−8.1	1967	−3, −18, 16	21, 32, 21	Chain A: ASP1009 GLU1010 TYR1017 ASP1019 THR1020 GLU1021 TYR1023 THR1025 ILE1028 GLY1029 HIS1030 LEU1031 LYS1034 ASP1069 VAL1102 PHE1103 GLN1104 MET1105 GLY1106 GLU1107 GLN1140 THR1141 ARG1144
C2	−9.4	910	9, 2, −47	21, 21, 21	Chain A: TYR25 VAL86 PHE87 SER90 PHE91 PHE94 HIS95 PHE106 LYS109 ILE110 VAL113 THR114 PHE117 LEU182 PHE183 PRO184 TRP258 VAL261 MET265 LYS278 PHE281 ALA282 SER285 CYS288
C3	−5.8	875	15, −8, −10	21, 21, 21	Chain A: HIS62 GLN63 ARG66 LYS67 PRO68 SER69 TYR70 ARG131 CYS134 PRO138 PRO139 TYR141 LYS142 ALA143 LEU145 THR146 ARG147 ALA235 ARG236 MET237 ARG238 LEU239 ASP240 LEU243 GLU305
C4	−6.1	423	10, 9, −18	21, 21, 21	Chain A: LEU49 GLU50 ASN51 VAL52 ALA53 VAL54 TYR56 LEU57 SER60 PRO296 VAL297 ILE298 ALA300 LEU301 ILE306 ARG307 SER309 ALA310 HIS311 CYS313 LEU314
C5	−5.4	312	18, 3, −57	21, 21, 21	Chain A: ALA19 PRO20 PRO21 MET22 LYS23 MET26 HIS98 VAL100 ASP101 SER102 LYS103 CYS174 PRO184 LEU185

**Table 4:** Molecular docking results of 1-trans-delta-9-tetrahydrocannabinol (THC) on CB1 receptor obtained from the CB-Dock2 server.

CurPocket ID	Vina Score (kcal/mol)	Cavity Volume (Å3)	Center (x, y, z)	Docking Size (x, y, z)	Contact Residues
C1	−11.2	3456	−39, −168, 310	29, 22, 22	Chain A: PHE108 ILE169 PHE170 SER173 PHE174 ASP176 PHE177 HIS178 ARG182 ASP184 PHE189 LYS192 LEU193 VAL196 THR197 PHE200 ILE267 PHE268 PRO269 TRP279 TRP356 LEU359 MET363 PHE379 SER383 CYS386
C2	−7.7	889	−41, −111, 245	22, 22, 22	Chain A: THR1012 ASN1014 SER1058 THR1059 TRP1060 GLY1061 ASP1062 SER1064 ILE1065 GLU1066 CYS1093 GLY1094 ASP1095 TRP1098 TYR1100 GLY1128 ASP1129 PRO1130
C3	−8.9	881	−46, −138, 299	31, 22, 22	Chain A: SER152 TYR153 HIS154 PHE155 ILE156 LEU209 ALA210 ALA211 ASP213 ARG214 TYR224 ILE227 VAL228 THR229 ARG230 LYS232 ALA233 ALA236 PHE237 TYR294 GLU340 LEU341 THR344 LEU345 ILE348 ILE396 TYR397 ALA398 ARG400 SER401 ARG405
C4	−6.0	395	−41, −135, 285	22, 22, 22	Chain A: ARG214 SER217 ILE218 PRO221 MET295 ILE297 LEU298 TRP299 LYS300 ALA301 HIS302 HIS304 ALA305 VAL306 MET337 ASP338 ILE339 GLU340 LEU341 ALA342 LYS343 THR344 LEU345 VAL346 ILE348 ILE396 TYR397 ARG400
C5	−5.7	236	−42, −132, 273	22, 22, 22	Chain A: ILE297 LEU298 TRP299 ALA301 HIS302 SER303 HIS304 ALA305 VAL306 ALA1002 LYS1003 ALA1004 ALA1029 GLY1030 TYR1031 GLU1032 VAL1033 ASP1034 GLY1049 PHE1050 ASP1051 LEU1052 ARG1145 ILE1148 MET337 ASP338 ILE339 ALA342

**Table 5:** Molecular docking results of 1-trans-delta-9-tetrahydrocannabinol (THC) on CB2 receptor obtained from the CB-Dock2 server.

CurPocket ID	Vina Score (kcal/mol)	Cavity Volume (Å3)	Center (x, y, z)	Docking Size (x, y, z)	Contact Residues
C1	−7.7	1967	−3, −18, 16	22, 32, 22	Chain A: ASP1009 GLU1010 TYR1017 LYS1018 ASP1019 THR1020 GLU1021 TYR1023 THR1025 ILE1028 GLY1029 HIS1030 LEU1031 LYS1034 ASP1069 ALA1072 ALA1073 VAL1102 PHE1103 GLN1104 MET1105 GLY1106 GLU1107 GLN1140 THR1141 ARG1144
C2	−11.5	910	9, 2, −47	22, 22, 22	Chain A: TYR25 PHE87 SER90 PHE91 PHE94 HIS95 PHE106 LYS109 ILE110 GLY111 VAL113 THR114 PHE117 LEU182 PHE183 PRO184 LEU191 TRP194 TRP258 VAL261 MET265 PHE281 ALA282 SER285 CYS288
C3	−5.9	875	15, −8, −10	22, 22, 22	Chain A: HIS62 GLN63 ARG66 LYS67 PRO68 SER69 TYR70 ARG131 CYS134 TYR141 LYS142 ALA143 LEU145 THR146 ARG147 ALA235 ARG236 MET237 ARG238 LEU239 ASP240 LEU243 GLU305
C4	−6.8	423	10, 9, −18	22, 22, 22	Chain A: LEU46 LEU49 GLU50 VAL52 ALA53 VAL54 TYR56 LEU57 SER60 PRO296 VAL297 TYR299 ALA300 LEU301 ILE306 ARG307 ALA310 CYS313 LEU314 ALA315
C5	−5.3	312	18, 3, −57	22, 22, 22	Chain A: ALA19 PRO20 PRO21 MET22 LYS23 MET26 HIS98 VAL100 ASP101 SER102 LYS103 PRO184 LEU185

**Table 6:** Comparison of the best CB-Dock2 molecular docking Vina scores (kcal/mol) of licofelone, 1-trans-delta-9-tetrahydrocannabinol (THC) and WIN 55,212-2 on CB1 and CB2 receptors.

Ligand	Vina Scores (kcal/mol)	
	CB1	CB2
Licofelone	−8.1	−9.4
THC	−11.2	−11.5
WIN 55,212-2	−10.6	−13.5

## Data Availability

Data will be made available on request.

## Supplementary Materials

The following supporting information can be downloaded at: https://www.mdpi.com/article/10.3390/biomedicines12071545/s1, Table S1: Number of animals used in each treatment group; Table S2: Molecular docking results of WIN 55212-2 on CB1 receptor obtained from the CB-Dock2 server; Table S3: Molecular docking results of WIN 55212-2 on CB2 receptor obtained from the CB-Dock2 server.
