# Supplementary material for: Licofelone, a Dual COX/LOX Inhibitor, Ameliorates Paclitaxel-Induced Mechanical Allodynia in Rats in a Cannabinoid Receptor-Dependent Manner
Source: Biomedicines. 2024 Jul 11;12(7):1545. doi: 10.3390/biomedicines12071545 (PMC11274467; doi:10.3390/biomedicines12071545)
Supplement: Supplementary file 1 [file biomedicines-12-01545-s001.zip › biomedicines-3027463-supplementary.pdf]

Supplementary Table S1. Number of animals used in each treatment group

**Group 1 Effects of licofelone on paclitaxel-induced mechanical allodynia.**	
**Treatment group**	**Number of animals**
Paclitaxel + vehicle	**8**
Paclitaxel + licofelone 12.5 mg/kg	**8**
Paclitaxel + licofelone 25 mg/kg	**6**
Paclitaxel + licofelone 50 mg/kg	**5**
Paclitaxel + licofelone 100 mg/kg	**5**
**Group 2 Effects of indomethacin plus minocycline combination (IPM) on paclitaxel-induced mechanical allodynia.**	
Vehicle	**8**
Paclitaxel + vehicle	**8**
Paclitaxel + indomethacin 10 mg/kg	**8**
Paclitaxel + minocycline 50 mg/kg	**8**
Paclitaxel + (Indomethacin 10 mg/kg + minocycline 50 mg/kg combination) IPM 60 mg/kg	**8**
**Group 3 Effects of cannabinoid receptor antagonists on indomethacin plus minocycline combination (IPM)'s antiallodynic activities**	
Vehicle	**8**
AM 251 3mg/kg	**7**
AM 630 3mg/kg	**7**
Indomethacin 10 mg/kg + minocycline 50mg/kg	**7**
AM 251 3mg/kg + indomethacin 10 mg/kg + minocycline 50mg/kg	**8**
AM 630 3mg/kg+ indomethacin 10 mg/kg + minocycline 50mg/kg	**8**
**Group 4 Effects of cannabinoid receptor antagonists on licofelone's antiallodynic activities**	
Vehicle	**5**
Licofelone 50mg/kg	**5**
AM 251 3mg/kg+ licofelone 50 mg/kg	**5**
AM 630 3mg/kg+ licofelone 50 mg/kg	**5**

Supplementary Table S2. Molecular docking results of WIN 55212-2 on CB1 receptor obtained from the CB-Dock2 server.

CurPocket ID	Vina score (kcal/mol)	Cavity volume (Å3)	Center (x, y, z)	Docking size (x, y, z)	Contact residues
C1	-10.6	3456	-39, -168, 310	29, 23, 23	Chain A: PHE108 VAL110 PHE170 SER173 PHE174 PHE177 HIS178 PHE189 LYS192 LEU193 VAL196 THR197 PHE200 ILE267 PHE268 PRO269 ILE271 TYR275 LEU276 TRP279 TRP356 LEU359 MET363 LYS376 PHE379 ALA380 SER383 CYS386
C2	-10.0	889	-41, -111, 245	23, 23, 23	Chain A: THR1012 ASN1014 SER1058 THR1059 TRP1060 GLY1061 ASP1062 CYS1093 GLY1094 ASP1095 TRP1098 TYR1100 CYS1102 ASP1127 GLY1128 ASP1129 PRO1130
C3	-9.7	881	-46, -138, 299	31, 23, 23	Chain A: ARG150 PRO151 SER152 TYR153 HIS154 PHE155 ILE156 LEU209 ALA210 ALA211 ASP213 ARG214 TYR224 VAL228 THR229 ARG230 ALA233 PHE237 TYR294 GLU340 LEU341 THR344 LEU345 ILE348 ILE396 TYR397 ALA398 ARG400 SER401 LYS402 ASP403
C4	-6.9	395	-41, -135, 285	23, 23, 23	Chain A: ARG214 SER217 ILE218 PRO221 LEU222 TYR224 LYS225 MET295 ILE297 LEU298 TRP299 LYS300 ALA301 HIS302 HIS304 ALA305 VAL306 MET337 ASP338 ILE339 LEU341 ALA342 LYS343 VAL346
C5	-6.5	236	-42, -132, 273	-6.5	Chain A: MET295 LEU298 TRP299 LYS300 ALA301 HIS302 SER303 HIS304 ALA305 VAL306 ALA1002 LYS1003 ALA1029 GLY1030 TYR1031 GLU1032 ASP1034 GLU1048 GLY1049 PHE1050 ASP1051 LEU1052 ARG1086 ARG1145 ALA1147 ILE1148 MET337 ASP338 ILE339 ALA342 LYS343 VAL346

Supplementary Table S3. Molecular docking results of WIN 55212-2 on CB2 receptor obtained from the CB-Dock2 server.

CurPocket ID	Vina score (kcal/mol)	Cavity volume (Å3)	Center (x, y, z)	Docking size (x, y, z)	Contact residues
C1	-9.3	1967	-3, -18, 16	23, 32, 23	Chain A: ASP1009 GLU1010 TYR1017 LYS1018 ASP1019 THR1020 GLU1021 TYR1023 THR1025 ILE1028 GLY1029 HIS1030 LEU1031 LYS1034 ASP1069 ALA1072 ALA1073 VAL1102 PHE1103 GLN1104 MET1105 GLY1106 GLU1107 THR1141 ASN1143 ARG1144 ARG1147
C2	-13.5	910	9, 2, -47	23, 23, 23	Chain A: TYR25 MET26 PHE87 SER90 PHE91 PHE94 HIS95 PHE106 LYS109 ILE110 GLY111 VAL113 THR114 PHE117 GLU181 LEU182 PHE183 PRO184 ILE186 TYR190 LEU191 TRP194 TRP258 VAL261 MET265 LYS278 PHE281 ALA282 SER285 CYS288
C3	-6.5	875	15, -8, -10	23, 23, 23	Chain A: HIS62 GLN63 ARG66 LYS67 PRO68 SER69 TYR70 ARG131 CYS134 PRO138 TYR141 LYS142 ALA143 LEU145 THR146 ARG147 ALA1159 TYR1160 ALA235 ARG236 MET237 ARG238 LEU239 ASP240 LEU243 GLU305
C4	-7.7	423	10, 9, -18	23, 23, 23	Chain A: LEU46 LEU49 GLU50 VAL52 ALA53 VAL54 TYR56 LEU57 PRO296 VAL297 ALA300 LEU301 ILE306 ARG307 ALA310 HIS311 CYS313 LEU314
C5	-6.6	312	18, 3, -57	23, 23, 23	Chain A: ALA19 PRO20 PRO21 MET22 LYS23 ASP24 MET26 HIS98 VAL100 ASP101 SER102 LYS103 LEU185
